# Reduction of Infantile Mortality

**Published:** 1914-06

**Authors:** 


**Reduction of Infantile Mortality.** W. H. Guilfoy, Registrar of Records in the N. Y. Dept. of Health, has prepared a pamphlet from the official statistics of the old city of N. Y., now the Boroughs of Manhattan and Bronx. In 1891, the population under five years was 188,703, the deaths 18,224. In 1913, the population was 333,595, the deaths 12,442. While the deaths have fluctuated somewhat, the first number is the maximum, the last the minimum of the entire series. The annual death rate has fallen from 96.5: 1000 children to 37.3. The deaths (likewise for children under five) in the summer months, June, July and August, range from the maximum of 6,612 in 1892 to the minimum of 3,261 in 1913. These deaths correspond to an annual mortality of 135.2: 1000 down to 38.8: 1000. Credit is given to the Nathan Strauss Pasteurized milk depots, instituted in 1892. Without wishing in any way to detract from the appreciation due to Mr. Strauss, we question whether the use of pasteurized milk has been the sole factor.

